# Small Infrared Target Detection by Region-Adaptive Clutter Rejection for Sea-Based Infrared Search and Track

**DOI:** 10.3390/s140713210

**Published:** 2014-07-22

**Authors:** Sungho Kim, Joohyoung Lee

**Affiliations:** 1 Yeungnam University 280 Daehak-Ro, Gyeongsan, Gyeongbuk 712-749, Korea; 2 Agency for Defense Development, 111 Sunam-dong, Daejeon 305-600, Korea; E-Mail: jhlee@add.re.kr

**Keywords:** IRST, small target, regional clutter, cloud, horizontal line, sea-glint, classification, background subtraction, temporal consistency

## Abstract

This paper presents a region-adaptive clutter rejection method for small target detection in sea-based infrared search and track. In the real world, clutter normally generates many false detections that impede the deployment of such detection systems. Incoming targets (missiles, boats, *etc.*) can be located in the sky, horizon and sea regions, which have different types of clutters, such as clouds, a horizontal line and sea-glint. The characteristics of regional clutter were analyzed after the geometrical analysis-based region segmentation. The false detections caused by cloud clutter were removed by the spatial attribute-based classification. Those by the horizontal line were removed using the heterogeneous background removal filter. False alarms by sun-glint were rejected using the temporal consistency filter, which is the most difficult part. The experimental results of the various cluttered background sequences show that the proposed region adaptive clutter rejection method produces fewer false alarms than that of the mean subtraction filter (MSF) with an acceptable degradation detection rate.

## 1. Introduction

**Figure 1.**
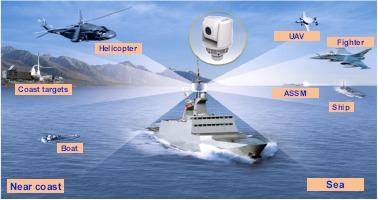
Operational concept of sea-based infrared search and track (IRST).

Sea-based infrared search and track (IRST) systems are wide field-of-view or omni-directional surveillance systems designed for autonomous search, detection, acquisition, track and designation of potential targets, as shown in Figure 1 [1,2]. The most important threats in sea-based IRST are incoming small targets, such as anti-ship sea-skimming missiles (ASSM) or asymmetric ships. In these applications, targets are typically unresolved and appear in the sky and sea backgrounds with a resolution of only a few pixels. Normally, a small infrared target's size is less than 100 pixels [3]. The important performance parameters of the target detection system consist of the radiant intensity of a target, detection distance, detection rate and false alarm rate. If the radiant intensity of a target and a minimal detection distance are determined, the detection algorithm should be able to detect true targets to satisfy the systems' detection rate and reject false targets as much as possible.

**Figure 2.**
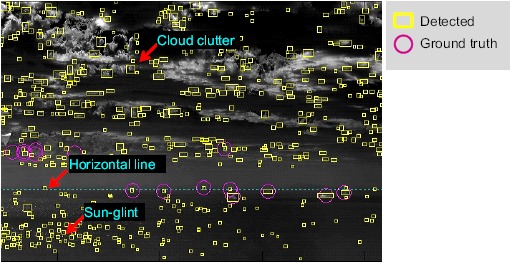
Problems of the conventional spatial filter-based, small target detection method. Many false detections are generated by regional clutter, such as clouds, horizon and sun-glint.

The detection of long-range, small targets is quite difficult, because of the small and dim target signal. The criteria of the detection rate can be achieved by lowering the detection threshold. On the other hand, such simple approaches lead to an increased number of false detections due to background clutters. Figure 2 shows the problems of the conventional small target detection method using the well-known modified mean subtraction filter (MMSF) [4]. The edge around cloud clutter can generate false detections. The horizontal edge line due to a heterogeneous background produces false detections. Finally, sun-glint has a similar shape (circular symmetry) to small targets and a high intensity value, which hinders true target detection. Such regional clutter produces many false alarms, which hinders true target detection.

This study examined how to make a small target detection method practical by reducing the number of false detections caused by different types of clutter, such as clouds in the sky, the edge line on the horizon and sun-glint in the sea surface region, in an integrated manner. According to geometric analysis, background images were segmented into the sky region, horizontal region and sea surface region. This paper proposes a region-adaptable clutter rejection scheme by careful observation and analysis of the clutter behavior. False detections around cloud clutter were removed by learning-based classification. The false detections around the horizon region were removed by subtracting the heterogeneous background. Finally, those around the sea surface region were removed by a temporal consistency filter. Therefore, the contributions from this study can be summarized as follows. The first contribution is the automatic region (sky-horizon-sea) segmentation by geometric analysis, which is an essential step in the clutter rejection system. The regions were segmented using the horizontal line estimated by the sensor pose-based prediction and image-based line fitting. The second contribution is the proposed region-adaptive false detection rejection scheme based on the analysis results. The third contribution is the demonstration of the proposed method using infrared test sequences by a comparison with the conventional detection method.

Section 2 reviews some related works on detecting small infrared targets focusing on the false alarm reduction aspect and analyzing the disadvantages of the related well-known methods of detecting small targets in heterogeneous backgrounds. Section 3 analyzes the target position in an infrared image based on the target type and incoming scenario. Section 4 introduces the overall system structure and presents the novel region adaptive clutter rejection methods. In Section 5, a range of performance evaluations and results are explained. Section 6 reports a discussion of the results with the conclusions.

## 2. Related Works in Terms of Clutter Rejection

Many studies have evaluated small infrared target detection methods over the past 20 years. This section reviews the related papers in terms of their use of information, such as target information, background information, visual context and decision information, to reduce the number of false alarms, as shown in Table 1, where the total sum of statistics is 100%. For example, a cause of false alarms due to clouds can be handled using the spatial information (14.2%) of the background cue and the shape information (5.8%) of the target cue. As a second example, a cause of false alarms due to sun-glint can be handled using motion information (3.5%) of the target cue, a high-level classifier (2.8%) of the decision cue, frequency information (2.2%) of target cue, multi-sensor fusion (2.1%) of the context cue or temporal information (1.5%) of the background cue. The following subsections introduce false alarm reducing methods and the related papers for the cloud clutter and sun-glint.

### 2.1. Related Studies on Cloud Clutter Rejection

Several studies have examined the removal or reduction of false detections caused by clouds. Their false alarm reduction strategies were strongly dependent on the situation. If there is any assumption, background subtraction can be a feasible approach. The background image can be estimated from an input image using spatial filters, such as the least mean square (LMS) filter [5–7], mean filter [8], median filter [9] and morphological filter (Top-hat) [10,11]. The LMS filter minimizes the difference between the input image and background image, which is estimated by the weighted average of the neighboring pixels. The mean filter can estimate the background by the Gaussian mean or simple moving average. The median filter is based on the order statistics. The median value can remove point-like targets effectively. The morphological opening filter can remove the specific shapes by erosion and dilation with a specific structural element. The mean filter-based target detection is computationally very simple, but sensitive to edge clutter. Target detection with non-linear filters, such as the median or morphology filter, shows low false alarms around the edge, but is computationally complex. Combinational filters, such as max-mean or max-median, can preserve the edge information of cloud and background structures [12]. A data fitting approach, which models the background as multi-dimensional parameters, has also been reported [13]. The super-resolution method is useful in a background estimation, which enhances small target detection [14]. The filtering process of localized directional Laplacian-of-Gaussian (LoG) filtering and the minimum selection can then remove false detection around cloud edges, maintaining a small target detection capability [15].

If a sensor platform is static, the information regarding the fast target motion is enhanced by removing the slowly moving cloud clutter. A well-known approach is the track-before-detect (TBD) method [16,17]. The concept is similar to that of the 3D matched filter. Dynamic programming (DP), which is a quick version of the traditional TBD method, achieves good performance in detecting dim targets [18,19]. The temporal profiles, including the mean and variance, at each pixel are effective in the detection of moving targets in slowly moving clouds [20–23]. Recently, the temporal contrast filter (TCF)-based method was developed to detect supersonic small infrared targets [24]. Accumulating the detection results of each frame makes it possible to detect moving targets [25]. The wide-to-exact search method was developed to enhance the speed of 3D matched filters [26]. Recently, an improved power-law-detector-based moving target detection method was presented; it was effective for image sequences that occur in heavy clutter [27].

Cloud clutter can also be reduced using decision methods. These decision methods need to determine that a probing region is a target. The hysteresis method has two thresholds. The first threshold is a very low value and is used to identify the candidate target regions. The second threshold possesses a relatively high value that depends on the operational requirements [28]. As information regarding the size becomes available, it is possible to remove large sun-glint and other large objects. Similar results can be obtained by applying an iterative threshold [29]. Statistics-based adaptive threshold methods, such as the constant false alarm rate (CFAR), are useful in a severely cluttered background [30,31]. The simplest classification method is the nearest neighbor classifier (NNC) algorithm, which uses only feature similarity [32]. In addition to NNC, there are model-based the Bayesian classifier [33], learning-based neural network, and support vector machine (SVM) [34] methods. Classification information can be useful for removing various clutter points.

### 2.2. Related Works on Sun-Glint Clutter Rejection

Sun-glint clutter can be rejected using the TBD methods mentioned above. These approaches, however, assume a high frame rate to reduce sun-glint. If the frame rate is approximately 1 Hz, a new approach should be developed.

On the other hand, frequency domain approaches can be useful for removing low frequency clutter. The 3D-FFT spectrum-based approach shows a possible research direction in the target detection [35]. The wavelet transform extracts the spatial frequency information in an image pyramid, which shows robustness in sun-glint environments [36–38]. The low-pass filter (LPF)-based approach can also be robust to sensor noise and sun-glint [39]. Recently, an adaptive high-pass filter (HPF) was proposed to reduce cloud and sun-glint clutter [40].

While the target is in motion, the previous frame is considered a background image. Therefore, a background estimation can be performed using a weighted autocorrelation matrix update using the recursive technique [41]. Static clutter can also be removed by the frame difference [42]. An advanced adaptive spatial-temporal filter derived by the multi-parametric approximation of clutter can achieve tremendous gain compared to that of the spatial filtering method [43]. Principal component analysis (PCA) for multi-frames can remove temporal noise, such as sun-glint [44].

The information fusion approach can be useful for reducing sun-glint. This includes the target-background context, multi-feature context, multi-band context and multi-classification context. Those visual contexts are implemented in the form of information fusion that leads to clutter reduction and high detection rates. The target-background context concomitantly enhances the target signature and reduces the background clutter, leading to a reduction of sun-glint clutter [45]. Multi-feature fusion can improve the detection rate of dim targets [46,47]. If spectral fusion, such as the ratio of mid-wave infrared and long-wave infrared or a combination of the detection results from both bands, is used, the sun-glint can be removed easily [48,49]. The voting of various classifiers can enhance the dim target detection rates [50].

## 3. Location Analysis of Incoming Targets

**Figure 3.**
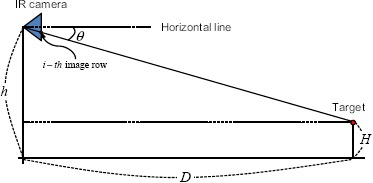
Simplified IRST projective geometry by assuming a flat surface. The triangle represents an IRST sensor, and the circular target is projected on the 1D infrared detector. The relationship between the target pixel position and target distance can be found using Equations (1)–(3).

**Figure 4.**
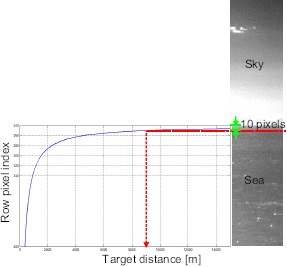
Analysis results for the target distance (*D*) and projected image position (*i*). (**Left**) The left graph represents the relationship between the target distance and target pixel location using Equation (3); (**Right**) while the right image is the corresponding example of the IR scene. If the sensor height is 20 m, the target height is 0 m and the minimal detection range is 6000 m, then the ship target is located 15 pixels below the horizontal line.

How can the target distance from a project target pixel be calculated? The target distance is a very important system parameter of IRST According to previous analysis, the projective relationship among the camera height (*h*), target distance (*D*), target height (*H*) and target positioning angle (*θ*) can be simplified as shown in Figure 3. In this scheme, the camera elevation angle (*α*) is assumed 0°. The target positioning angle can be estimated by the camera height and target distance, as expressed in Equation (1). If it is assumed that the camera's field of view (FOV) is 6° and the size of the IR detector is 480, the projected target position (*i* – *th* image row) can be calculated using Equation (2). Because this study was interested in the relationship between the row image position and target distance, the final projective relation can be obtained as Equation (3), which is derived from Equations (1) and (2). If it is assumed that the camera height is 20 m, the ship height is 0 m and the minimal target detection range is 9000 m, the ship target is projected into 10 pixels just below the horizontal line, as shown in Figure 4. In the case of a sea-skimming missile, of which the whole normal flying height is 200 m, the projected image is located just 10 pixels above the horizontal line at the minimal 8000-m detection. If the height (*H*) of the ASSM is lower than the camera height (*h*), the target is located around the horizontal line. As it approaches the camera, it appears on the sea surface. From such geometrical analysis related to the target types, it can be concluded that the distant targets are located around the horizontal line (±20 pixels centered on the horizontal line at 5000-m detection), and relatively close targets exist in the sky region or sea surface region. Therefore, it is necessary to segment an input image into the sky region, horizontal region and sea surface region.

(1) $$\theta =\tan^{-1}\left(\frac{h-H}{D}\right)\times \frac{180}{\pi }$$

(2) $$i=(\theta +3)\times \frac{480}{6}$$

(3) $$D=\frac{h-H}{\tan\left(\left(\frac{i}{80}-3\right)\times \frac{\pi }{180}\right)}$$

## 4. Proposed Small Target Detection with Region-Wise Clutter Rejection

**Figure 5.**
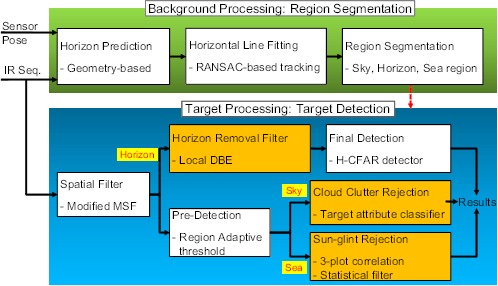
Overall small target detection flow based on region segmentation and region-specific clutter rejection.

The proposed small target detection consists of background processing and target processing, as shown in Figure 5. The background processing module segments an input image into sky, horizon and sea region using the sensor pose information and image processing. The target processing module finds the candidate targets using a spatial filter and rejects any false alarms caused by background clutter using carefully-designed methods. The spatial filter (modified mean subtraction filter (MSF)) is commonly used in the entire region. Horizontal line clutter is estimated by a local directional background estimation (DBE) and removed. Small targets in the horizontal region are detected by the hysteresis threshold-based constant false alarm detector (H-CFAR). The candidate targets in the sky and sea regions are found by pre-detection. False detections in the sky region are generated by clouds. Therefore, the target attribute-based classifier can reject false detections caused by cloud clutter. False detections by sea-glint in the sea region are rejected by a three-plot correlation and statistical filter. The following subsections introduce details of the region segmentation, removal of the horizontal line clutter in the horizon region, removal of cloud clutter in the sky region and removal of sea-glints in the sea region.

### 4.1. Geometry and Image-Based Region Segmentation

**Figure 6.**
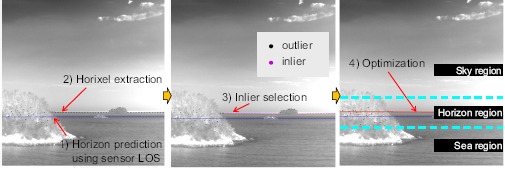
Region segmentation flow by horizontal line prediction and optimization.

Horizontal information is very important, because it can provide a region segmentation cue. Therefore, region segmentation can be conducted in the following four steps: (1) horizon prediction using sensor LOS, (2) horizon pixel (horixel) extraction, (3) inlier selection and (4) horizon optimization and region segmentation, as shown in Figure 6. The horizontal location can be predicted using sensor pose information. The next step is the optimal horizon tracking in a video sequence. Given an input frame, the horixels are extracted using a column directional gradient and max selection. The inlier horixels are identified using the robust line fitting method of RANSAC [51]. The important role of RANSAC is to find the inlier indices of the true horixels. Based on the inlier index, the total least squares optimization can detect the final horizon stably. Because the inlier horixels are identified through the process, horizon tracking is conducted using horixel extraction and optimization. The inlier detection block is activated in the beginning and statistically to adapt to environmental changes.

**Figure 7.**
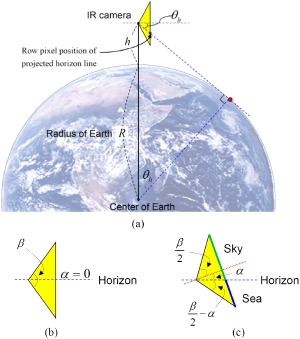
Geometry of the sea-based IRST system. (**a**) Relationship between the sensor height and horizontal line; (**b**) camera geometry with the field of view and elevation angle (*α* = 0); (**c**) approximated position of the horizontal line when the elevation angle is α.

Sensor pose-based horizon prediction: If it is assumed that an IR camera has a height (*h*), elevation angle (*α*, assuming 0° for easy analysis) and Earth radius (*R*), then the geometric relations can be depicted as shown in Figure 7a. The projected horizontal line in any image can be found by calculating the angle (*θ_H_*), as shown in Equation (4). A real IRST sensor can change the elevation angle, which alters the location of the horizontal line in the image domain. If the elevation angle of a camera is given as *α* and the field of view (FOV) of the sensor is given as *β*, then the angle of the sky region (*θ_sky_*) is determined by Equation (5). If the elevation angle (*α*) is smaller than *θ_H_* − *β*/2, the sensor can only observe the sea region. Therefore, the angle of the sky region (*θ_sky_*) is zero. Similarly, other cases can be analyzed. The angle of the sea region (*θ_sea_*) is determined as, *θ_sea_* = *β* − *θ_sky_*. As the sky-sea region segmentation ratio is determined by *tanθ_sea_*/*tanθ_sky_*, the final horizontal line (*H_prior_*) is calculated using Equation (6). If it is assumed that the image height is 1280 pixels, the vertical field of view is 20°, the sensor height is 20 *m* and the elevation angle is 5°, then the prediction horizontal line (*H_prior_*) is located as shown in Figure 6 (the blue dotted line in the first image).

(4) $$\theta_{H}=-{\text{cos}}^{-1}\left(\frac{R}{R+h}\right)$$

(5) $$\theta_{\text{sky}}=\{\begin{array}{cc} 0 & \text{if}\;\alpha <\theta_{H}-\beta /2\\ \beta & \text{if}\;\alpha >\theta_{H}+\beta /2\\ \alpha -\theta_{H}+\beta /2 & \text{else} \end{array}$$

(6) $$H_{\text{prior}}=\text{ImageHeight}*\frac{\tan\theta_{\text{sky}}}{\tan\theta_{\text{sky}}+\tan\theta_{\text{sea}}}$$

Horixel extraction: Given a predicted horizon, as shown in Figure 6 (dotted blue line), a search boundary is set. The sampling interval is then defined to reduce the computational complexity. For each sample position, the column direction gradient filter is conducted using the derivative of the Gaussian kernel. The horixels close to a predicted horizon are then extracted by max selection. Figure 6 (dotted black line in the first image) shows the extracted horixels.

Inlier detection using RANSAC: In a sea environment, the horizon is occluded frequently by islands, coasts and clouds. Therefore, a robust horizon estimation method, such as RANSAC, is needed. Basically, the RANSAC algorithm chooses two horixels and predicts the horizon line. The algorithm then checks the line fitting and inliers. After a number of iterations, a horizon line parameter with the largest inliers is selected. Figure 6 (the second image) shows the inlier detection results using a RANSAC method. Note that the inliers and outliers are classified almost correctly. The inlier indices are used to optimize line fitting and horizon tracking.

SVD-based optimization and tracking: The last step is to refine horizon parameters using a total least squares fit of a givenset of inlier horixels. The fitting process is as follows. First, the inlier horixels are normalized, and a singular value decomposition (SVD) is conducted [52]. The horizon direction is selected by an eigenvector with the smallest eigenvalue. Figure 6 (the last image) shows the horizon optimization results for an image occluded by near island and remote island. The horizontal area is enlarged to show the results. Horizon tracking is done by a horixel extraction and SVD-based optimization with the inlier indices. RANSAC-based initialization is activated statistically.

### 4.2. Horizon Region: Removal of Horizontal Line Clutter

**Figure 8.**
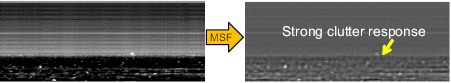
A 2D local mean subtraction filter (MSF) produces a strong response around the horizontal line where the heterogeneous regions exist.

The mean subtraction filter (MSF)-based small target detection method is based on the 2D mean filter [8]. The 2D mean filter is used to estimate the local background with a window size of 5 × 5 or 7 × 7. The MSF-based approach has been deployed in several countries, because of its simplicity and high detection capability of small targets [8,53,54]. A modified MSF (M-MSF) is used to enhance the signal-to-noise ratio using a pre-smoothing input image. On the other hand, the 2D local mean subtraction filter produces a strong response around the horizontal line, which prevents target detection or produces false detection, as shown in Figure 8. If a global threshold or constant false alarm rate (CFAR) detection are applied, the true target pixels are buried in the horizontal line pixel, which leads to the failure of horizontal target detection.

**Figure 9.**
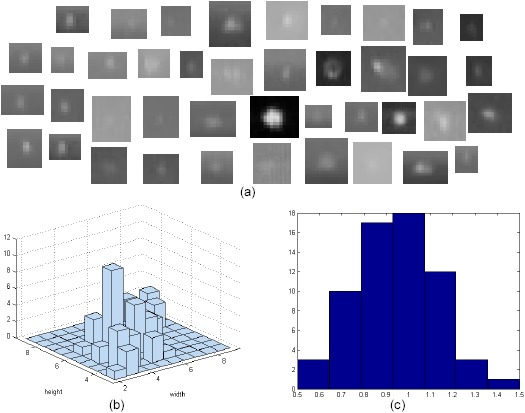
Observations of real infrared targets. (**a**) Examples of small infrared targets; (**b**) histograms of target size in terms of the width and height; (**c**) histograms of the aspect ratio (height/width).

According to real target observations, the targets have Gaussian shapes, as shown in Figure 9. Figure 9 presents partial target examples, the distribution of the target size (width, height) and the aspect ratio of observed targets, respectively. According to the statistics, the targets have blob-like structures (mean size: (width = 5.1 pixels, height = 5.4 pixel) with a standard deviation (width = 1.7, height = 1.4) and aspect ratio of ~1). Note that the sizes include very low intensity pixels belonging to the target region. Therefore, a Gaussian-like filter is introduced. This idea is similar to the matched filter theory. If the filter coefficients are the same as the target shape, the the maximum signal-to-noise ratio is achieved. In this paper, the 2D Gaussian filter coefficients was set to *G*_3×3_(*x*, *y*) = [0.1 0.11 0.1; 0.11 0.16 0.11; 0.1 0.11 0.1], which is generated by a 2D Gaussian function with a kernel size of three and a standard deviation of 1.4. The filter coefficients should be changed according the specific target applications.

**Figure 10.**
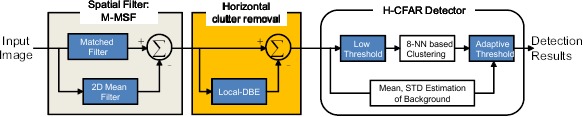
Proposed small target detection and horizontal line clutter removal in the horizon region.

Therefore, the proposed M-MSF is conducted as follows (see Figure 10). An input image (*I*(*x*, *y*)) is pre-filtered using the proposed filter coefficients (*G*_3×3_(*x*, *y*)) to enhance the signal-to-clutter ratio (SCR), as shown in Equation (7) using the matched filter (MF). The SCR is defined as (max target signal—background intensity)/(standard deviation of background). Simultaneously, the background image (*I_BG_*(*x*, *y*)) is estimated by a 7 × 7 moving average kernel (*MA*_7×7_(*x*, *y*)), as expressed in Equation (8). The pre-filtered image is subtracted by the background image, which produces an image (*I_M_*_–_*_MSF_*(*x*, *y*)), as shown in Figure 9. The number of false detections is reduced with the same thresholds compared to that of the previous method. Therefore, the proposed M-MSF can improve the previous 2D local MSF in terms of false detections and the SCR of the true target.

(7) $$I_{\text{MF}}(x,y)=I(x,y)*G_{3\times 3}(x,y)$$

(8) $$I_{\text{BG}}(x,y)=I(x,y)*{\text{MA}}_{7\times 7}(x,y)$$

(9) $$I_{M-\text{MSF}}(x,y)=I_{\text{MF}}(x,y)-I_{\text{BG}}(x,y)$$

The horizontal region should be processed further to remove the structural clutter, such as the horizontal line. After applying M-MSF, a SCR-improved image can be achieved. This suggests that the salt-and-pepper noise is reduced and the target signal is enhanced. The local directional background estimation (L-DBE) is applied directly to the horizontal region of the M-MSF result. In the scan-based sensor of IRST, the row pixels show similar responses, particularly around the horizontal region. Estimating the background along the scan direction for each row is reasonable. For each row, the number of target pixels is much smaller than that of the background pixels. The row directional background can be estimated based on this observation. The target pixel values are considered as outliers, whereas the background pixel values are regarded as inliers. The proposed L-DBE (*I_L_*_–_*_DBE_*(*x*, *y*)) is defined as Equation (10), where the tab size is 2*n* + 1. A 1D local median filter is used to handle the image tilt error. Because a normal target size is approximately five pixels, the filter size (2*n* + 1) should be five to 10 times larger than the target size to achieve a stable background estimation. In the test environment, *n* = 35 to solve both the stable background estimation and image tilt problems.

**Figure 11.**
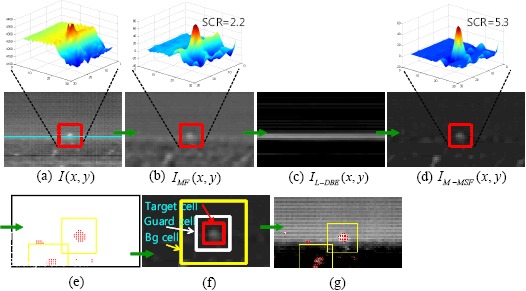
Visualization of horizontal clutter removal and detection flow: (**a**) input image; (**b**) matched filter; (**c**) horizontal line clutter estimation; (**d**) modified (M)-MSF results; (**e**) pre-thresholding; (**f**) signal-to-clutter ratio (SCR) computation region; and (**g**) final detection results using the SCR threshold.

Figure 11 shows the overall procedures of the spatial filtering process for the horizontal region introduced in this section. The input of the L-DBRFis the output (*I_M_*_–_*_MSF_*(*x*, *y*)) of the previous filter stage from which the directional background (*I_L_*_–_*_DBE_*(*x*, *y*)) is estimated. The output (*I_L_*_–_*_DBRF_*(*x*, *y*)) of the consecutive filter can be calculated using Equation (11). Note the improvement of the SCR during the application of M-MSF and L-DBRF. Because the horizontal background clutter is estimated and removed in the L-DBRF stage, the clutter noise is reduced, leading to an enhancement of the SCR calculation.

(10) $$I_{L-\text{DBE}}(x,y)=\text{median}\{I_{M-\text{MSF}}(x-n,y),I_{M-\text{MSF}}(x-n+1,y),\cdots ,I_{M-\text{MSF}}(x,y),\cdots ,I_{M-\text{MSF}}(x+n-1,y),I_{M-\text{MSF}}(x+n,y)\}$$

(11) $$I_{L-\text{DBRF}}(x,y)=I_{M-\text{MSF}}(x,y)-I_{L-\text{DBE}}(x,y)$$

The last step of small target detection in the horizon region is how to decide which pixels correspond to the target pixels. This paper proposes a new region hysteresis-threshold-based constant false alarm (H-CFAR) detector, as depicted in Figure 11e–g. A global threshold can be used to detect a possible target. On the other hand, it cannot work properly where different dense clutter exists. The global threshold-based detection scheme can be modified by incorporating the region segmentation information (sky, horizon, sea) to adapt to the properties of different backgrounds. In addition, a local background adaptive threshold, called the CFAR, can handle the clutter problem, because the threshold values are adaptive to the density of background clutter to produce constant false alarms. Directly applying the CFAR to each pixel is time consuming, because it needs to calculate the mean and standard deviation of the background pixels. The key idea is to use two region-adaptive thresholds in a hysteresis threshold framework (H-CFAR). As shown in Figure 11e, the pre-threshold is selected to be as low as possible. At the same time, the regional properties should be considered properly to find the candidate target region. The eight-nearest neighbor (8-NN)-based clustering method is used to group the detected pixels. The sizes of the possible targets can be estimated by 8-NN clustering. The probing region is divided into the target cell, guard cell and background cell, as depicted in Figure 11f. A target cell size is the same as the results of Threshold 1 with clustering. The background cell size is determined to be three- to four-times the size of the target cell. The guard cell is just a blank region that is not used in both regions and set as a two- or three-pixel gap. The second threshold (*k_region_*) in the CFAR can detect the final targets. *μ_BG_* and *σ_BG_* represent the average and standard deviation of the background region, respectively. *k_region_* denotes the region-dependent second threshold used to control the detection rate and false alarm rate. Normally, the threshold values have the following order: *k_horizon_* <*k_sky_*<*k_sea_*. Figure 11g presents the final detection results (called plots in IRST) by applying Equation (12) to Figure 11d.

(12) $$\begin{array}{l} \text{A probing region is a target if}\\ \text{SCR}(x,y)=\frac{\left|T_{\text{max}}-\mu_{\text{BG}}\right|}{\sigma_{\text{BG}}}>k_{\text{region}} \end{array}$$

### 4.3. Sky Region: Removal of Cloud Clutter

**Figure 12.**
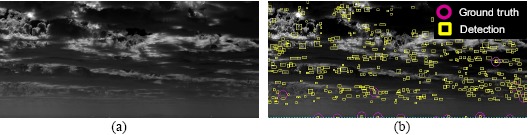
Problems of false alarms caused by cloud clutter: (**a**) original infrared image; (**b**) M-MSF + hysteresis threshold-based constant false alarm rate (H-CFAR) detection.

The detection results shown in Figure 12b can be obtained by applying the H-CFAR detector after spatial filtering to an IRST image, where many false detections caused by the strong cloud clutter exist for a given test image, as shown in Figure 12a. Machine learning approaches are applied to this problem. A classifier divides the correct targets and clutter points in the feature space. The simplest method is the nearest neighbor classifier (NNC) algorithm, which uses only the feature similarity [32]. In addition to NNC, there are the model-based Bayesian classifier [33], learning-based neural network and support vector machine (SVM) [34] methods. Classification information can be useful for removing various clutter points. On the other hand, it is difficult to apply these classification methods, because the targets are very small, resulting in little information being available. This paper proposes eight small target feature types and analyzes them in terms of discrimination. In this study, machine learning-based clutter rejection schemes were developed based on this feature analysis.

**Figure 13.**
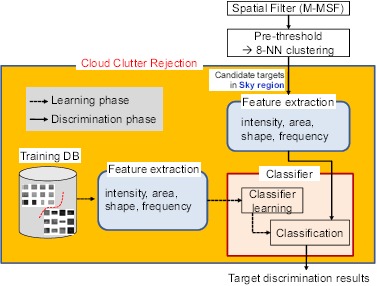
Overall flow of the target discrimination.

As shown in Figure 13, the cloud clutter rejection system consists of a learning phase and a discrimination phase. In the learning phase, a training database (DB) is prepared automatically using the target detection algorithm and ground truth information. The classifiers are learned using the extracted features. In the discrimination phase, the features are extracted by probing the target regions, which are obtained by the spatial filter (M-MSF) and 8-NN clustering after a pre-threshold; the final target discrimination is performed by the learned classifier.

Small infrared targets are normally small bright blobs of fewer than 100 pixels; extracting informative features from point-like target images is quite difficult. In this study, the standard deviation, ranked-fill-ratio, second-order moment, area, size ratio, rotational size variation, frequency energy and average distance methods were considered. In advance, a filtered database was considered to inspect the features.

The first feature (standard deviation) is a simple standard deviation of the image intensity for a considered region, as defined by Equation (13). *I*(*i*) denotes the intensity at the *i*-th pixels; *N* denotes the total number of pixels, and *μ* is the average intensity.

(13) $$\sigma =\sqrt{\frac{\sum_{i=1}^{N}{\left(I(i)-\mu \right)}^{2}}{N}}$$

The second feature (ranked-fill-ratio)) considers the ratio between the *K* brightest pixels and the total intensity, as defined in Equation (14). The targets normally have higher values than the clutter, because targets are observed as a hot spot on a cold background.

(14) $$\eta =\frac{\sum_{j\in K}I(j)}{\sum_{i}I(i)}$$

The third feature (second order moment) considers the second image moment as defined in Equation (15).

(15) $$m_{22}=\frac{\sum_{x}\sum_{y}{\left(x-\mu_{x}\right)}^{2}{\left(y-\mu_{y}\right)}^{2}I(x,y)}{\sum_{i}\sum_{j}I(i,j)}$$

The following five features are basically extracted from the target region: In the fourth feature (area), a black and white target region is obtained by applying Otsu's method, which chooses the threshold to minimize the intraclass variance of the black and white pixels [55]. Given a gray image *I*(*i*), the segmented target region is denoted as *R*(*i*). This feature can be calculated using the following equation:

(16) $$a=\sum_{i}R(i)$$

The fifth feature (Size Ratio) considers the target size ratio. If the target width is denoted as *l_W_* and the target height is expressed as *l_H_*, then the ratio can be defined as:

(17) $$S_{\text{ratio}}=\frac{l_{H}}{l_{W}}$$

The sixth feature (rotational size variation) is based on the rotational size profile (*L*(*i*)). A target size profile is generated by rotating the region. Therefore, the rotational size profile reflects the target shape. The profile is uniform if a small target has a circular blob, whereas it is similar to a cosine curve if it has a rectangular shape. The rotational size profile can be quantified using the standard deviation of the curve, as defined in Equation (18).

(18) $$\sigma_{L}=\sqrt{\frac{\sum_{i=1}^{N}{\left(L(i)-\mu_{L}\right)}^{2}}{N}}$$

The seventh feature regards the frequency energy and is obtained by applying a fast Fourier transform (*FFT*) to the rotational size profile (*L*(*i*)):

(19) $$\begin{array}{l} M(k)=\text{FFT}(L(i)-\mu_{L}),\\ f_{\text{energy}}=\overset{M}{\underset{k=1}{\text{∑}}}\frac{{\left|M(k)\right|}^{2}}{M} \end{array}$$

The last feature is the mean distance. If a region consists of *N* pixels and the region center is (*μ_x_*, *μ_y_*), the average Euclidean distance can be calculated using the following equation:

(20) $$d=\frac{\sum_{i=1}^{N}\sqrt{{\left(x(i)-\mu_{x}\right)}^{2}+{\left(y(i)-\mu_{y}\right)}^{2}}}{N}$$

**Figure 14.**
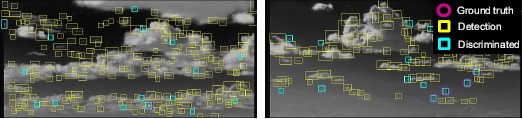
Cloud clutter rejection examples using the proposed feature and AdaBoost classifier.

This section thus far discussed the feature extraction methods to discriminate infrared small targets and cloud clutters. The remainder of the process is the selection of the optimal classifier. In this study, AdaBoost was chosen, because it can select the features suitable for discriminating true targets. The SVM method considers multi-dimensional feature vectors and finds the support vectors using a kernel recipe. AdaBoost, on the other hand, uses simple weak classifiers (*h_i_*), as well as the weighted sum of weak classifiers, which leads to a strong classifier, as expressed in Equation (21). In this study, the weak classifiers are just simple threshold-based binary decisions for individual feature space. Figure 14 presents examples of cloud clutter rejection using the proposed method. Note that the proposed scheme can remove false detections by cloud clutter.

(21) $$H_{\text{strong}}(\text{x})=\mathrm{sign}\left(\sum_{i=1}^{N}\alpha_{i}h_{i}(\text{x})\right)$$

### 4.4. Sea Region: Removal of Sea-Glint

**Figure 15.**
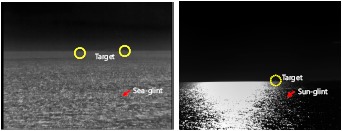
Example of sun-glint in the infrared search and track system.

Sea-glint makes the detection of small targets in the sea region a challenging problem, as shown in Figure 15. The dotted circle indicates the true target, and the arrow indicates the sun-glint. The irradiated target energy is quite small, due to scattering and absorption through the atmosphere. This leads to a dim target, whose signal-to-noise ratio (SNR) is quite low. The dim targets are composed of 2–10 pixels. The target intensity level is similar to that of the neighboring pixels. Furthermore, sun-glint has a similar shape (circular symmetry), like small targets, and a high intensity value, which hinders true target detection.

**Figure 16.**
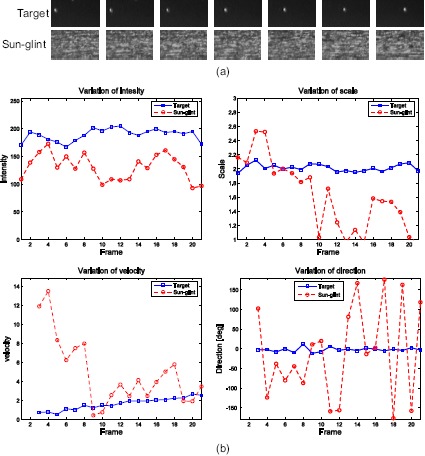
Observation of a target and sun-glint: (**a**) sequence of a target and sun-glint; (**b**) observation results in terms of the intensity, scale, velocity and moving direction.

Why is the detection of a small target very difficult? If each frame is observed, as shown in Figure 16a, the targets and sun-glint have small bright spots. Therefore, spatial shape information cannot discriminate the true targets and sun-glint. On the other hand, if targets and sun-glint are observed in the temporal domain, observation results can be obtained in terms of intensity, scale, velocity and moving direction, as shown in Figure 16b. The key property is consistency. The targets show a consistent intensity, scale, velocity and direction compared to sun-glint.

**Figure 17.**
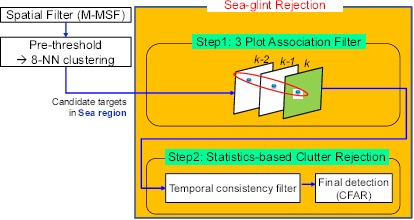
Proposed small target detection system. The system consists of a geometric sea region extraction part, spatial filtering part, three-plot correlation-based temporal filter part and statistics-based clutter rejection part.

According to the survey, there have been few studies on small target detection in a dense sun-glint clutter environment. A single spatial filter cannot remove sun-glint clutter as the signatures of a true target, and sea-glint has a quite similar shape with circular symmetry. A conventional motion cue cannot be utilized, as the target may be stationary and the frame rate is very low. Therefore, this paper proposed a hybrid method by making a compromise for the spatial filter approach and temporal approach, known as the separate spatio-temporal filtering method based on an attribute-based plot association. The plot indicates only the candidate target in IRST. The underlying assumption is that a true target behaves like an outlier in both the spatial and temporal domains. The behavior of sun-glint is random, but that of the targets is consistent. Such a concept is used in the design of spatial and temporal filters. Figure 17 represents the proposed target system based on these concepts. The top component level consists of a plot association-based temporal filtering part and a statistics-based clutter rejection part, given the candidate targets extracted by pre-detection using M-MSF and pre-thresholding. In the temporal filtering part, this paper proposes a three-plot association filter based on the target attributes for data association. After a three-plot association, the sea-glint clutter is reduced further using a temporal consistency filter and constant false alarm (CFAR) detection method.

The next step is to produce a group of plots, called the three-plot correlation or association to remove sun-glint. In general, this can be considered a target tracking problem, like Bayesian filtering, shown in Equation (22). *x_k_* denotes the target position to be estimated; *z_k_* denotes the observed target position, and *Z_k_* denotes the observation sequence data up to the *k* – *th* frame. *p*(*x_k_*|*Z_k_*_–1_) acts as a prior target position estimated from the previous frames. Data association should be conducted to link a target track and an observation in measurement, *p*(*z_k_*|*x_k_*). This approach is focused on estimating target position using a large amount of frame data.

(22) $$p(x_{k}|Z_{k})=\frac{p(z_{k}|x_{k})p(x_{k}|Z_{k-1})}{p(z_{k}|Z_{k-1})}$$

where:

(23) $$p(x_{k}|Z_{k-1})=\int_{x_{k-1}}p(x_{k}|x_{k-1})p(x_{k-1}|Z_{k-1})$$

**Figure 18.**
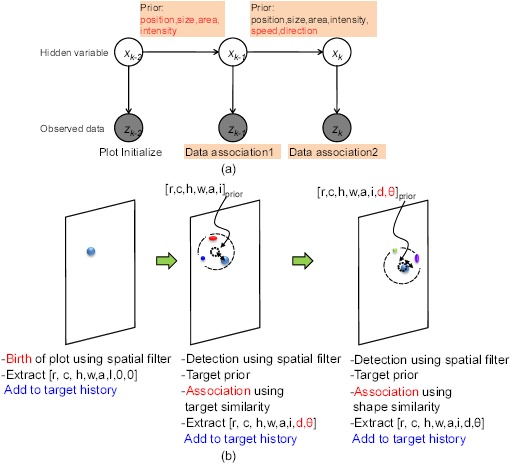
Concept of temporal filter using data association: (**a**) Graphical model-based representation of a three-plot correlation filter; (**b**) implementation procedures. The first frame is used to generate initial plots without prior knowledge. In the second frame, the prior target attribute is used for data association. In the third frame, the prior motion is also used during data association.

In the target detection problem, the focus is on how to remove sun-glint within three frames (system requirement), but leave the tracking of the targets relatively unscathed. As mentioned earlier, the basic assumption is that targets behave as outliers compared to the sun-glint. This suggests that sun-glint behaves randomly, but true targets behave consistently. Therefore, the false alarms caused by the sun-glint can be removed through the three-plot correlation using a graphical model. Figure 18a shows the basic concept of a three-plot correlation using a graphical model. The white circle denotes the hidden variable, and the gray circle denotes the detected target data. The correlation is concerned only with a prior prediction and data association given in three consecutive frames. Figure 18b shows a corresponding three-plot correlation process. The first frame is used to generate an initial plot, whose attribute is $F_{t-2}={\left[\text{row}(r),\text{column}(c),\text{height}(h),\text{width}(w),\text{area}(a),\text{intensity}(i),0,0\right]}_{\text{prior}}^{k-2}$. Given this information, this plot can be associated with the new plot in the second frame. The association is conducted by finding the maximum target similarity using the previous attribute information. The feature distance measure that is proposed in Equation (24) is used. This can measure the shape distance by summing the differences in the heights, widths, areas and intensities between the associating targets. The target motion, such as moving distance (*d*) and moving direction (*θ*), can be found during the consecutive association. The previous unassociated plot (*k* – 2) is removed automatically, and the currently unassociated plot (k – 1) generates a new plot. Given this attribute ( $F={\left[r,c,h,w,a,i,d,\theta \right]}_{\text{prior}}^{k-1}$), the second plot can be associated with the third plot using the target attribute and the target motion prediction. If the three consecutive plot attributes are collected, a statistics-based clutter rejection is conducted, which is explained in the following subsection.

(24) $$S_{\text{dist}}(F_{t-1},F_{t})=\left|h_{t-1}-h_{t}\right|+\left|w_{t-1}-w_{t}\right|+\left|a_{t-1}-a_{t}\right|+\left|i_{t-1}-i_{t}\right|$$

**Figure 19.**
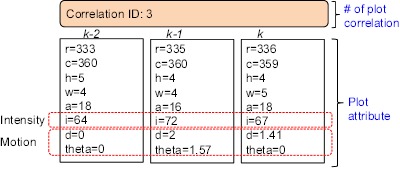
Attributes of the three-plot correlation and temporal behavior data of intensity and motion used for a statistics-based clutter rejection.

The previous three-plot correlation method checks only the shape similarity of the associating targets. If the temporal behavior, such as the intensity statistics and motion statistics, is considered, the sun-glint can be removed further for the three correlated plots (*correlation ID* = 3), as shown in Figure 16. Given the plot attributes, as shown in Figure 19, the intensity consistency filter (*C_I_*) and motion consistency filter (*C_M_*) can be applied using Equations (25) and (26), respectively. *σ* denotes the standard deviation, and *d_Th_* denotes the distance threshold of the target motion. Although the number of data points is just three, these filters are powerful for rejecting sun-glint. The standard deviation of both the plot intensity and plot motion are used. On the other hand, the standard deviation of the motion direction is considered only if the motion is large enough to avoid the image noise effect (e.g., *d_Th_* > 2 pixels).

(25) $$C_{I}=\sigma ([i_{k-2},i_{k-1},i_{k}])$$

(26) $$\begin{array}{l} \text{if}\;\;\;d_{k}>d_{\text{Th}}\\ C_{M}=\sigma ([\theta_{k-2},\theta_{k-1},\theta_{k}]) \end{array}$$

**Figure 20.**
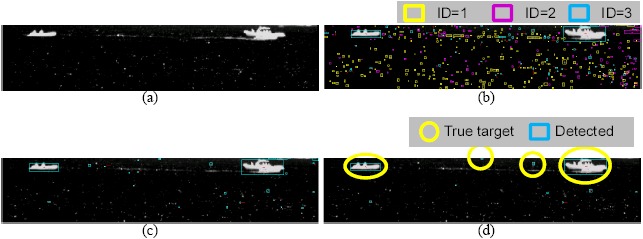
Example of the target detection flow for a sea regional infrared image. (**a**) Test image; (**b**) three-plot correlation results (ID denotes the number of plot correlation); (**c**) temporal filter-based clutter reduction; (**d**) final detection using the H-CFAR method. The circles represent true targets, and squares represent detected targets.

To explain the proposed detection system depicted in Figure 17, this paper presents the overall processing flows with the related results for a standard test image, as shown in Figure 20. The test IR image (Figure 20a) has possible targets on the sea. Figure 20b represents the detection results using a three-plot correlation filter. The ID indicates the number of correlations. For this process, M-MSF and pre-thresholding are used for spatial candidate target detection. Figure 20c represents the results of a statistics-based temporal filtering. Figure 20d shows the targets finally detected using the H-CFAR method. Table 2 summarizes the clutter reduction rate for this test sequence. The proposed three-plot correlation filter can reduce 50% of clutters. Through the temporal filter and CFAR detection, we can achieve up to 97.7% of clutter rejection, while detecting the true targets.

## 5. Experimental Results

**Figure 21.**
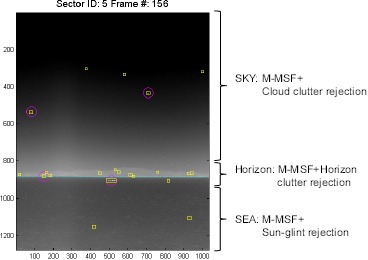
Proposed region-adaptive, small target detection and clutter rejection scheme.

This paper introduced details of the proposed region segmentation by horizon detection, horizontal line clutter rejection, cloud clutter rejection and sun-glint rejection, as shown in Figure 21. In this section, each proposed item was evaluated by comparing the conventional methods, and then, the integrated method was applied to test sequences.

### 5.1. Evaluation of Horizontal Line Detection

**Figure 22.**
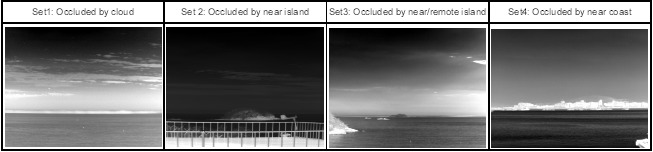
Composition of the test database for horizontal line detection.

Four kinds of test sequences were prepared, as shown in Figure 22, to validate the robustness of the proposed method. Set 1 is remote sea images occluded by a strong cloud. Set 2 is occluded by the island nearby, which occupies 1/3 of the horizon length. Set 3 has nearby islands and a remote island. The last one, Set 4, has a coast nearby, in which boats and buildings occlude the horizon.

**Figure 23.**
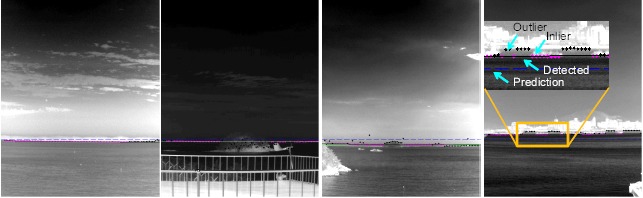
Examples of horizon detection for the test Sets 1–4.

A detected horizon is declared to be a correct detection if the line fitting error is within one pixel on average. The ground truth of the horizon location was prepared by a manual inspection. The original test sets had almost no sensor noise. Therefore, artificial sensor tilt noise and horizon location noise by the ±0.5° and ±3.0 pixels, respectively, were generated by the uniform distributionfor that range. Table 3 lists the overall experimental results. The proposed method detected the horizons correctly for the noiseless sequence data. In the case of the noisy data, only one frame of Set 4 showed incorrect horizon detection. Figure 23 shows the sampled horizontal detection results for the noise-added sequences. The dotted blue lines denote the horizon prediction by sensor LOS. The solid black or white line denotes the optimal horizon. The magenta dots denote the inlier horixels extracted by RANSAC. Note that the horizon lines are detected robustly, regardless of the occlusion types under sensor noise.

### 5.2. Evaluation of Horizontal Clutter Rejection

**Figure 24.**
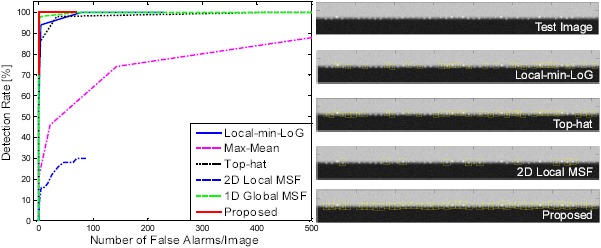
ROC curves and related detection examples. (**a**) ROC curves of three different spatial filters; (**b**) detection examples with thresholds of zero false alarms.

In an evaluation of horizontal clutter rejection, the detection rate and false alarms per image were compared to evaluate the detection performance according to the different spatial filter types. As initial experiments, a synthetic image was prepared by background modeling and target modeling. The background image had a sky region and a background region with an intensity difference of 100 gray values. The horizontal line was smoothed further column-wise using a Gaussian filter. Fifty targets were generated with different sizes and difference SCR values. Those targets were inserted around the horizontal line, as shown in the top of Figure 24b. The targets generated have a size range of (3 × 3) to (10 × 10) and an SCR range of 0.97 to 1.95. The ROC curve metric was used to evaluate the filtering method for this test image. The pre-threshold (*Th_pre_*) was set as five, and the H-CFAR threshold (*k*) was changed from one to 20. Figure 24a shows the evaluation results. The results with a 2D Local MSF [8] show a very small ROC region and a relatively low detection rate. The max-mean filter [12] also produces a poor ROC area. The 1D Global MSF-based method showed much larger ROC region, but produced many false detections (more than 4000 false alarms with *k* = 1) with a small threshold value. Recent methods, local-min-LoG and Top-hat filter, showed good performances [15,56]. In contrast, the proposed method (horizontal clutter rejection (L-DBRF) after M-MSF) showed an ideal ROC curve pattern. Note that the maximum number of false alarms was just 70 with *k* = 1. Figure 24b shows the target detection results using three types of spatial filters. The H-CFAR thresholds were tuned to make zero false alarms. The proposed method could detect all of the targets successfully.

**Figure 25.**
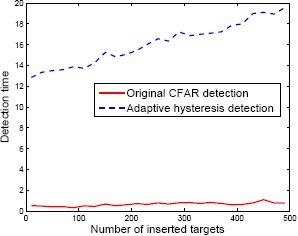
Processing time of the decision methods: CFAR *vs.* H-CFAR (adaptive hysteresis detection).

In the next evaluation, the target decision methods were compared. The original CFAR detector probes all of the pixels above the noise level. On the other hand, the proposed decision method (H-CFAR) uses an adaptive hysteresis threshold consisting of a small threshold for candidate detection and a CFAR threshold for the final decision. A test image consists of a different number of synthetic targets from 10 to 490. Figure 25 presents the comparison results. The processing time of the original CFAR detection took approximately 16.1 s, which increased with increasing numbers of targets. In contrast, the processing time of the proposed detection method took approximately 0.65 s and increased slightly with increasing number of targets. Both decision methods showed similar detection results.

### 5.3. Evaluation of Cloud Clutter Rejection

**Figure 26.**
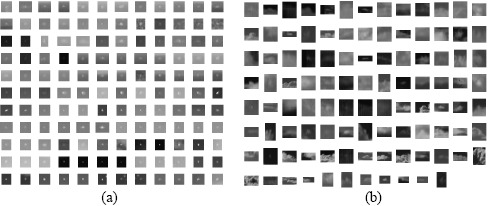
Target and clutter database for classifier learning: (**a**) target chips; (**b**) clutter chips.

A sufficiently large data set is important for ensuring successful learning for cloud clutter rejection. In this study, 136 real target images were collected using either a mid-wave infrared (MWIR) camera or a long-wave infrared (LWIR) camera. The target images were acquired by real airplanes, such as the KT-1, F-5 and F-16. The cloud clutter database was prepared using the detection algorithms introduced in the previous section. Figure 26 provides examples of the target and clutter images.

The naive Bayes, SVM and AdaBoost classifiers were compared in the evaluation. The training samples were selected randomly, and the remaining samples were used for the test set. The average detection rate (DR) and false alarm rate (FAR) were evaluated over 100 iterations. Table 4 lists the results. Although the naive Bayes method produced a low FAR, it had a relatively low DR. The DR is more important in target discrimination, because true targets need to be detected. The SVM classifier produced an improved DR, but had a high FAR. The AdaBoost classifier (29 weak classifiers after learning) produced an improved DR with a lower FAR than that found for the SVM. Therefore, AdaBoost was selected as a classifier to reject cloud clutter in the sky region.

### 5.4. Evaluation of Sea-Glint Rejection

**Figure 27.**
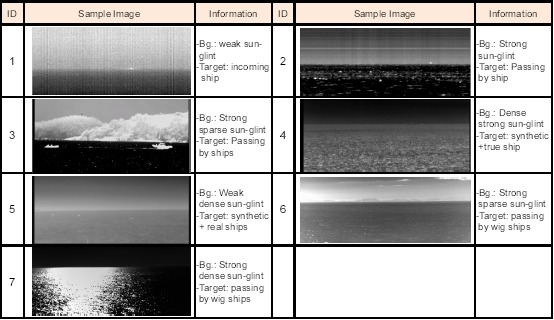
Composition of the test database.

A set of sea-based IRST images were prepared to test and evaluate the proposed method. Figure 27 summarizes seven kinds of test sequences that were acquired by mid-wave infrared (MWIR) cameras. Set 1 has weak sun-glints with an incoming ship scenario. Set 2 has strong sun-glint with ships passing by. Set 3 has strong sparse sun-glints with large ships near the coast. Set 4 has dense strong sun-glints with a synthetic incoming target and far away true targets. Set 5 has weak dense sun-glint with a synthetic incoming target and several real ships. Set 6 has strong sparse sun-glint with WIGships passing by in a remote coastal environment. Set 7 has strong dense sun-glint with WIG ships passing by. Each image set was used selectively, depending on the evaluation.

**Figure 28.**
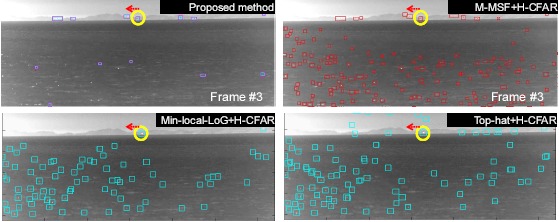
Target detection comparison between the proposed method and the baseline method for test Set 6.

The proposed sea-glint rejection method was compared with the baseline methods ((M-MSF, Min-local-LoG [15], Top-hat [56]) + H-CFAR detection) for five kinds of test sets (Set 4, Set 5, Set 6, Set 7). The detection rate (DR) and number of false alarms (FAR) per image were used as the comparison measures. For a fair comparison, the detection rates were fixed for each data set by tuning threshold values. For each test set, the ground truths were prepared manually. Table 5 lists the overall performance results for the five different test sets in terms of false alarm rate (number of false detections per image). The proposed method showed the same detection rate as the baseline method, but it produced fewer (approximately one- to 16-times fewer) false alarms than the baseline methods. Figure 28 shows the detection results for test Set 6, which had a real target (WIG ship) passing by a remote coast. In the proposed method, the squares denote the final detection by removing the edge targets. Note that the baseline methods produced a large number of false alarms around the sun-glint. According to the results, the proposed method (3 plot correlation + attribute filter) could detect the true targets robustly and produce a small number of false alarms in the sea-glint region.

### 5.5. Integrated Evaluation of the Proposed Method

**Figure 29.**
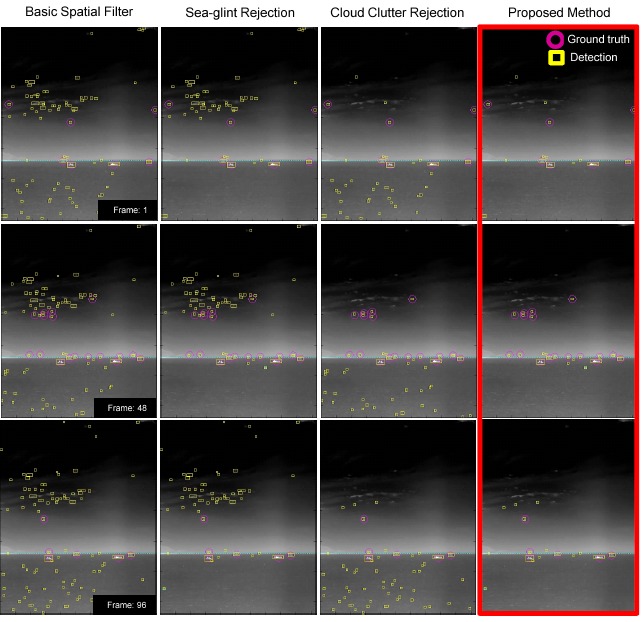
System performance comparison results by applying the clutter rejection methods.

As a final evaluation, the test sequence consisted of five sectors with 156 frames (1280 × 1024). A number of synthetic targets were generated using the method reported by Kim *et al.* [60]. The test sets consisted of cloud clutter and sea-glint. Table 6 lists the overall evaluation results depending on the clutter rejection schemes in terms of the detection rate and number of false alarms per frame. The basic spatial filter means the (M-MSF + L-DBRF) + H-CFAR detector. The basic one denotes M-MSF + pre-thresholding. The proposed method (region-wise clutter rejection) reduced the number of false detections by a factor of 2.5 to 9.4 per image, depending on the sector type by the clutter rejection schemes, with just a 0.1%–0.8% degradation in the detection rate. Figure 29 gives examples of the clutter rejection effects on the Sector 2 DB. Note that the false detections in the cloudy sky region and in the sea-glint region were removed almost completely by the proposed method, while still maintaining target detection.

## 6. Conclusions

Reducing the number of false detections caused by clutter in small infrared target detection is quite challenging due to the point-like target nature. Clutters have different natures depending on the types, such as horizontal line clutter, cloud clutter in the sky and sea-glint in the sea. This paper presented a region segmentation method based on horizontal line detection using both the sensor pose information and image processing. In the horizontal region, the process of the local directional background removal filter (L-DBRF) after the modified mean subtraction filter (M-MSF) can reject the horizontal line clutter and achieve a high detection rate with few false alarms per image. In the sky region, the AdaBoost discriminative learning method was proposed to remove cloud clutter based on the target attribute feature, such as intensity, area, frequency, *etc*. According to the results of the AdaBoost-based target discrimination method on the test sequence, a false alarm reduction was achieved with only a small amount of degradation in the detection rate. In the sea region, separate spatio-temporal filtering was proposed to reject sea-glint. The temporal filter after a three plot correlation could reduce the sun-glint further. Through experimental comparisons, the proposed method was found to be robust for the detection of targets in a strong sun-glint environment using a low frame rate infrared camera, regardless of the target motion. In the final test, the proposed integrated clutter rejection scheme can effectively reduce the number of false detections by a factor of 2.5 to 9.4 with just 0.1%–0.8% degradation in the detection rate. Therefore, the proposed scheme is expected to be useful for sea-based infrared search and tracking systems.

## Figures and Tables

**Table 1.** Statistics of research papers in terms of the causes of false alarms and the methods of overcoming them (%).

Causes of False Alarms		Dim	Noise	Background Clutter			Similar Object	
				**Cloud**	**Glint**	Ground	Bird	Buoy
Target cue	Intensity	0	0	1.4	0	0	0	0
	Shape	0	0.7	5.8	0.7	1.4	0	0
	Motion	5.7	2.1	5.8	**3.5**	3.5	2.1	2.1
	Distance	0	0	1.4	0	0	1.5	0
	Freq.	0	0.7	1.4	2.2	0	0	0
Background cue	Spatial	0	0	**14.2**	0	14.2	0	0
	Temporal	0.7	0	0	1.5	1.4	0	0
Context cue	Region	0.7	0	0.7	0.7	2.1	0	0
	Fusion	4.3	0	0.7	2.1	2.1	0	0
Decision cue	Threshold	1.4	0	0.7	0.7	2.8	0	0
	Classifier	0.7	0.7	1.4	2.8	1.4	0	0

**Table 2.** Clutter reduction performance for each spatio-temporal processing module.

Processing	No. of Plots	Clutter Reduction Rate (%)
1-plot correlation	807	0.0
3-plot correlation	399	50.5
Temporal filter	289	64.2
CFAR detection	18	97.7

**Table 3.** Detection rate (DR) of the horizon for the noiseless data and noisy data.

Test Set	DR w/o Noise (%)	DR with Noise
Set 1	100 (20/20)	100 (20/20)
Set 2	100 (35/35)	100 (35/35)
Set 3	100 (35/35)	100 (35/35)
Set 4	100 (30/30)	97 (29/30)

**Table 4.** Performance of the: (**a**) naive Bayes, (**b**) SVM and (**c**) AdaBoost classifiers in terms of the detection rate (DR) and false alarm rate (FAR).

Measure	Naive Bayes [57]	SVM [58]	AdaBoost [59]
DR (%)	84.07	86.56	88.80
FAR (%)	6.03	13.58	8.70

**Table 5.** Statistical performance comparisons of the proposed method and baseline method. DR denotes the detection rate, and FAR denotes the number of false alarms per image. DB, database.

Test DB	DR	FAR (number/image)			

		Proposed	M-MSF	Min-local-LoG[15]	Top-hat [56]

Set 3 (strong, sparse)	94.3 (83/88)	**12**	24	20	40
Set 4 (strong, dense)	97.6 (121/124)	**2**	8	13	16
Set 5 (weak, dense)	99.2 (119/120)	**1**	16	9	16
Set 6 (strong, sparse)	98.6 (72/73)	**22**	99	74	102
Set 7 (strong, dense)	94.7 (71/75)	**18**	75	70	95

**Table 6.** Comparison of clutter rejection performance.

Test Set	Method	DR (%)	FAR (number/frame)
Sector 1	Basic spatial filter	99.8 (601/602)	272.0 (42,483/156)
	Basic + Cloud clutter reject	99.0 (596/602)	204.5 (31,909/156)
	Basic + Sun-glint reject	99.8 (601/602)	154.7 (24,136/156)
	Proposed	**99.0** (596/602)	**87.2** (13,607/156)

Sector 2	Basic spatial filter	99.8 (1576/1478)	80.6 (12,589/156)
	Basic + Cloud clutter reject	99.2 (1467/1478)	39.3 (6146/156)
	Basic + Sun-glint reject	99.8 (1576/1478)	49.8 (7769/156)
	Proposed	**99.2** (1467/1478)	**8.5** (1326/156)

Sector 3	Basic spatial filter	99.9 (1407/1422)	19.4 (3039/156)
	Basic + Cloud clutter reject	98.4 (1399/1422)	16.2 (2521/156)
	Basic + Sun-glint reject	98.9 (1407/1422)	7.7 (1206/156)
	Proposed	**99.2** (1399/1422)	**4.4** (688/156)

Sector 4	Basic spatial filter	99.4 (1356/1363)	24.4 (3816/156)
	Basic + Cloud clutter reject	99.3 (1353/1363)	21.6 (3376/156)
	Basic + Sun-glint reject	99.4 (1356/1363)	9.8 (1530/156)
	Proposed	**99.3** (1353/1363)	**6.9** (1089/156)

Sector 5	Basic spatial filter	99.7 (1079/1082	32.0 (4999/156)
	Basic + Cloud clutter reject	99.3 (1074/1082)	30.4 (4745/156)
	Basic + Sun-glint reject	99.7 (1079/1082)	14.3 (2233/156)
	Proposed	**99.3** (1074/1082)	**12.6** (1979/156)
